# Two-dimensional speckle tracking echocardiography demonstrates improved myocardial function after intravenous infusion of bone marrow mesenchymal stem in the X-Linked muscular dystrophy mice

**DOI:** 10.1186/s12872-022-02886-1

**Published:** 2022-11-03

**Authors:** Xiao Liu, Shixiang Yao, Min Pan, Yingying Cai, Weihui Shentu, Wenqian Cai, Hongkui Yu

**Affiliations:** 1 Department of Ultrasonography, Shenzhen Hospital of Guangzhou University of Chinese Medicine (Fu-tian), Shenzhen, Guangdong China; 2 Department of Ultrasonography, Guangzhou Women and Children’s Medical Center, Guangzhou Medical University, Guangzhou, Guangdong China; 3 Heart Center, Institute of Pediatrics, Guangzhou Women and Children’s Medical Center, Guangzhou Medical University, Guangzhou, Guangdong China; 4 Department of Ultrasonography, Shenzhen Children’s Hospital, Shenzhen, Guangdong China

**Keywords:** Duchenne muscular dystrophy, Bone marrow mesenchymal stem cells, Speckle tracking echocardiography, Strain, Mdx

## Abstract

**Background:**

Bone marrow mesenchymal stem cells (BMSCs) are commonly used in regenerative medicine. However, it is not clear whether transplantation of BMSCs can improve cardiac function of the X-Linked Muscular Dystrophy Mice (mdx) and how to detect it. We aimed to investigate the role of speckle tracking echocardiography (STE) in detecting cardiac function of the BMSCs-transplanted mdx in comparison with the untreated mdx.

**Methods:**

The experimental mice were divided into the BMSCs-transplanted mdx, untreated mdx, and control mice groups (n = 6 per group). The BMSCs were transplanted via tail vein injections into a subset of mdx at 20 weeks of age. After four weeks, the cardiac functional parameters of all the mice in the 3 groups were analyzed by echocardiography. Then, all the mice were sacrificed, and the cardiac tissues were harvested and analyzed by immunofluorescence. The serum biochemical parameters were also analyzed to determine the beneficial effects of BMSCs transplantation.

**Results:**

Traditional echocardiography parameters did not show statistically significant differences after BMSCs transplantation for the three groups of mice. In comparison with the control group, mdx showed significantly lower left ventricular (LV) STE parameters in both the long-axis and short-axis LV images (*P* < 0.05). However, BMSCs-transplanted mdx showed improvements in several STE parameters including significant increases in a few STE parameters (*P* < 0.05). Immunofluorescence staining of the myocardium tissues showed statistically significant differences between the mdx and the control mice (*P* < 0.05), and the mdx transplanted with BMSCs demonstrated significantly improvement compared with the untreated mdx (*P* < 0.05).

**Conclusion:**

This study demonstrated that the early reduction in the LV systolic and diastolic function in the mdx were accurately detected by STE. Furthermore, our study demonstrated that the transplantation of BMSCs significantly improved myocardial function in the mdx.

## Background

Duchenne muscular dystrophy (DMD) is the most common X linked recessive myopathy with an incidence rate of 1/3000–5000 in live male births [1]. It is caused by frame shift or nonsense mutations in the gene encoding dystrophin. This causes complete or partial deletion of dystrophin, which plays an essential role in maintaining the integrity of the skeletal and cardiac muscle membranes. Therefore, DMD is characterized by muscle fiber degeneration and necrosis. The onset of DMD symptoms occur between 3 and 5 years of age and are mainly presented as progressive weakness and atrophy of the skeletal muscles throughout the body [2]. The mortality of DMD patients is due to respiratory and circulatory failure. Despite significant increase in the lifespan of DMD patients due to improvements in care and treatment in the last decade, heart failure is the main cause of death in these patients [3].

Currently, there is no effective cure for DMD. Clinical treatment with glucocorticoids delays the disease progression but does not alter the outcome of the disease [4]. Long-term treatment requires correction of the gene defect or compensation of the dystrophin gene function. Therefore, gene therapy and cell therapy has attracted significant research in this area. Gene therapy strategies such as gene replacement therapy, gene editing, exon skipping techniques, and stop codon read-through have shown promising results [5–7]. However, optimal results have been lacking because of low transfection rates, poor stability of the transgene, and single treatment gene locus [8, 9].

Bone marrow mesenchymal stem cells (BMSCs) are early progenitor cells in the mesoderm developmental pathway that can differentiate into multiple cellular lineages and tissues including the muscle tissue. Mesenchymal stem cells (MSCs) are commonly used for clinical repair and cell transplantation therapy of diseased tissues in the human body, and have been used for tissue engineering studies in the recent years [10]. MSCs are easy to obtain, culture and amplify in vitro, and easy to transfect and express foreign or recombinant genes. Due to the low immunogenicity, the transplantation rejection rates of MSCs are low [11]. Therefore, MSCs are ideal transplantation cells for the treatment of DMD. Previous studies have shown that intravenous transplantation of BMSCs improves muscle regeneration and motor function in the DMD model mice [12, 13].

However, it is not known if cardiac dysfunction can be ameliorated by stem cell therapy. Early detection of myocardial dysfunction in the subclinical phase may facilitate timely intervention [14]. Hence, it is essential to characterize imaging modalities that can detect early cardiac dysfunction in the DMD patients. Echocardiography is one of the commonly used imaging modalities for the assessment of left ventricular (LV) function. Speckle tracking echocardiography (STE) is a new technique that analyzes motion of acoustic reflections and STE parameters have been validated as treatment outcome biomarkers in the small animal models [15]. Early impaired cardiac function has been successfully detected in puppies, mice, and children using STE and LV strain assessments [16–18].

The X-linked muscular dystrophy mouse (mdx) is a well-established mouse model of DMD and has been frequently used in the pathological, diagnostic, and therapeutic studies of DMD. The mdx harbor a recessive X-linked sporadic point mutation in exon 23 of the dystrophin gene and show similar pathological changes as the DMD patients [19, 20]. Therefore, in the present study, we investigated the efficacy of 2D STE imaging in detecting cardiac function changes and the expression of dystrophin after the intravenous transplantation of BMSCs in the mdx mice.

## Methods

### Mice and BMSCs

The animal experiments were approved by the Institutional Animal Care and Use Ethics Committee of Guangzhou Medical University (Guangzhou, China, Ethical No. G2022-103). Twelve-week-old mdx (C57BL/10ScSnJGpt-Dmd^em3Cd4^/Gpt) and the corresponding wild type C57BL/6J mice were purchased from Gem Pharmatech Co., Ltd (Nanjing, China). C57BL/6 mouse-derived bone marrow mesenchymal stem cells (BMSCs) were purchased from Linyun Biotechnology Co., Ltd (Guangzhou, China).

### Mice grouping and BMSCs transplantation protocol

**Fig. 1:**
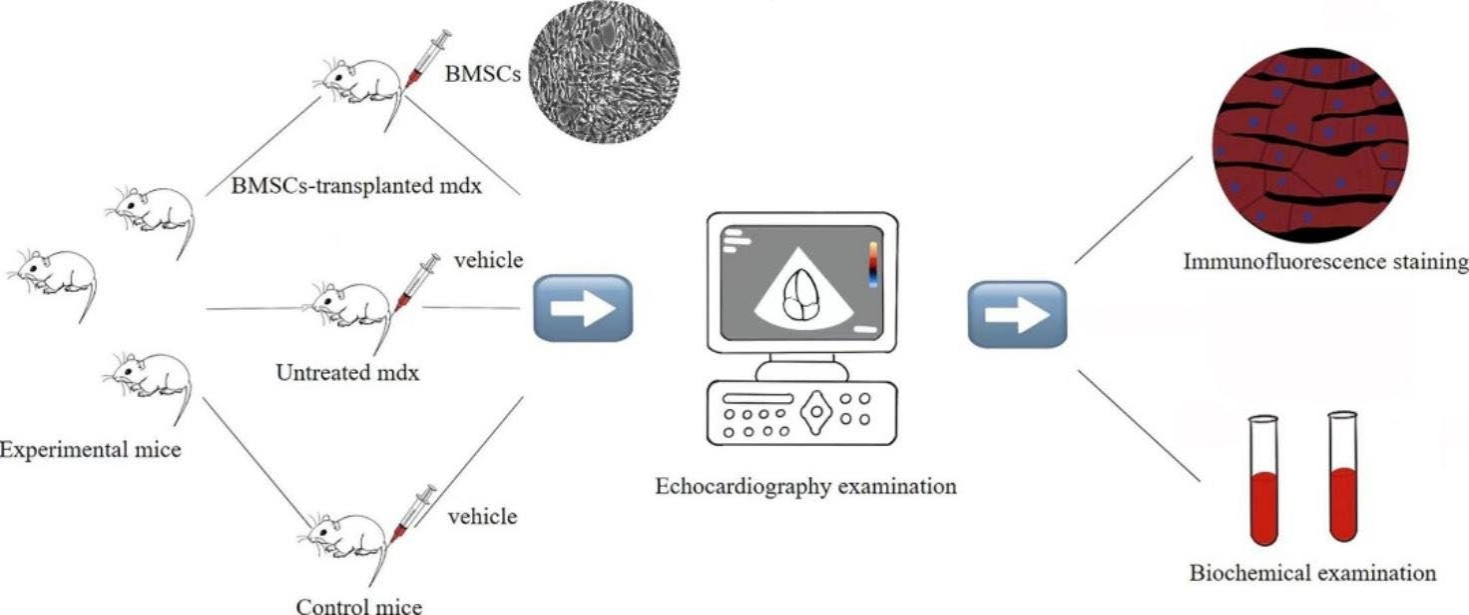
Study protocol

The experimental mice were divided into the following three groups with six mice each: Group 1, BMSCs-transplanted mdx; Group 2, untreated mdx; and Group 3, control mice. After 2 months of adaptive feeding, at 20 weeks of age, 5 × 10^5^ BMSCs (9th passage) were injected via the tail vein into each of the six mdx in the group 1. The group 2 and group 3 mice were injected with the vehicle (phosphate buffered saline, PBS) via the tail vein. The health status (weight, appearance, activity) of all the mice was analyzed once every week after the injections. After four weeks, cardiac function was evaluated by echocardiography. Then, all the mice were sacrificed and examined by histopathology (Fig. 1).

### Echocardiography

A high-frequency ultrasound system (Vevo 3100; VisualSonics, Toronto, ON, Canada) equipped with a single-crystal mechanical transducer (RMV 707B) and a central frequency of 30 MHz (15–45 MHz) was used to acquire the ultrasound images. Briefly, the mice were anesthetized with 3% inhaled isoflurane initially mixed with 100% oxygen and then maintained at 1.5% oxygen. The mice were laid in a supine position on a heated platform. The legs were taped to the electrocardiographic electrodes for recording. The chest hair was removed with a chemical hair remover before imaging.

M-mode echocardiography was performed to evaluate the cardiac morphology and function in the left ventricular (LV) long-axis view at the midpapillary muscle level. The M-mode gate was set in the middle between the two papillary muscles to measure the ejection fraction (EF) and fractional shortening (FS). LV end-diastolic dimension (LVEDD) and heart rate (HR) values were also estimated from the M-mode images.

For the STE examinations, short-axis two-dimension mode cine loops were acquired at the mid-level (midpapillary muscle level), and the long-axis cine loops were recorded by placing the scan head along the left sternal border for a clear view of the apex and the LV outflow tract. The frame rates of the regions of interest (ROIs) were adjusted up to 120 frames/sec. The image gain and other parameters were adjusted carefully to delineate all the myocardial segments. All the images were acquired for at least three successive and stable cardiac cycles. The raw data was stored digitally in a hard drive for offline analysis.

**Fig. 2:**
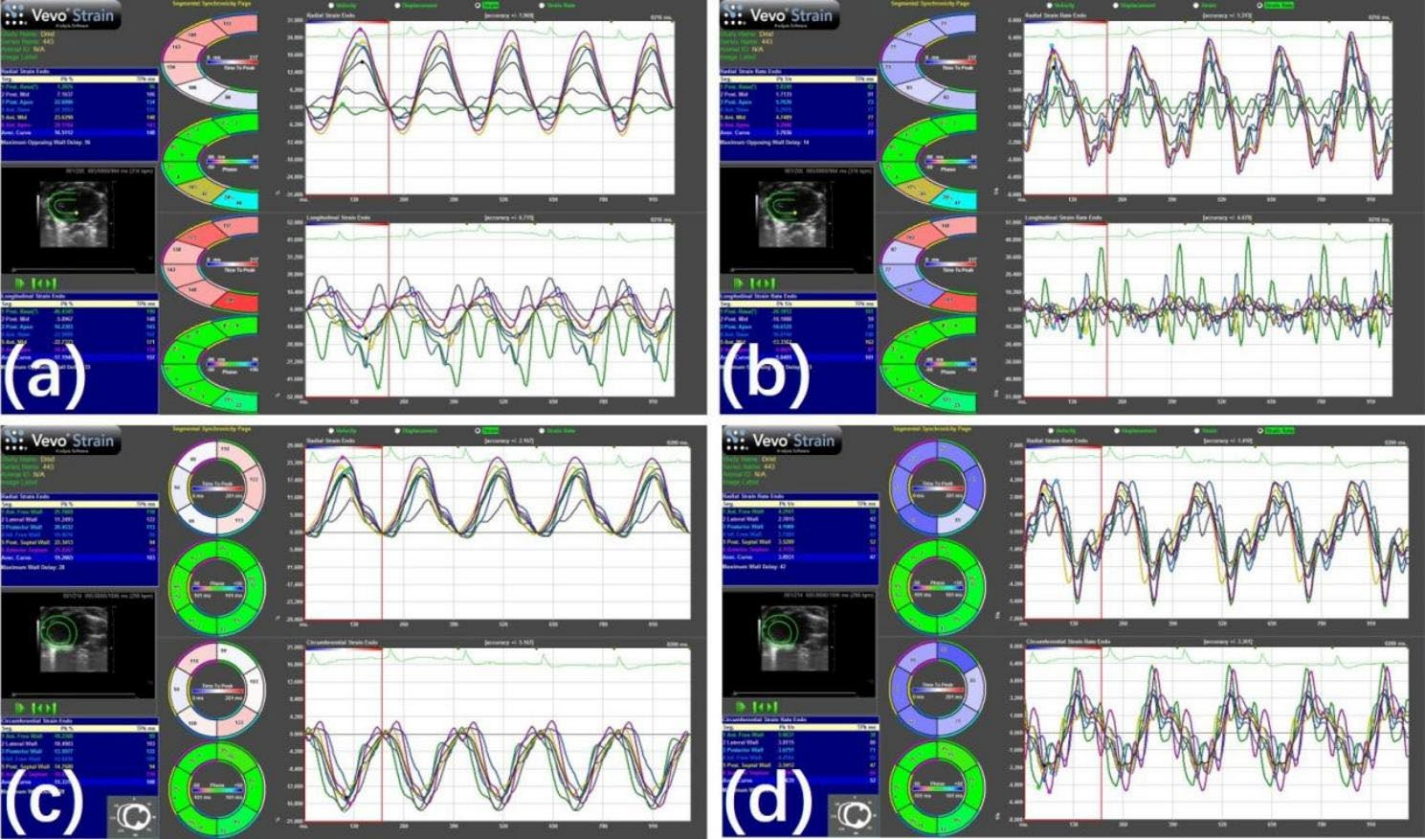
Strain and strain rate analyses of LV long-axis and short -axis view in an untreated mdx. (a) radial and longitudinal strain analyses of LV long-axis view. (b) radial and longitudinal strain rate analyses of LV long-axis view. (c) radial and circumferential strain analyses of LV short-axis view. (d) radial and circumferential strain rate analyses of LV short-axis view. Layout of each figure: Left column, top: analysis report; middle: semiautomated identification of long-axis (a and b) or short-axis (c and d) LV wall; bottom, analysis report. Middle column: time to peak analysis. Right column, curves of strains and strain rates. Each line represents the strain or strain rate of a segment of the left ventricle

STE analysis was performed using the 17 TomTec Image Arena 4.0 software (TomTec Imaging Systems, Unterschleissheim, Germany). Strain and strain rate (SR) were estimated by manual tracing and editing of the ROIs and semi-automated tracking of the entire LV myocardial deformation from the epicardial to the endocardial border. The software automatically divided the long-axis LV images into six segments, namely, basal anterior, mid anterior, apical anterior, apical posterior, mid posterior, and basal posterior. The software also divided the short-axis images into six segments, namely, anterior, anterior septum, posterior septum, inferior, posterior, and lateral. Strain measurements in each segment were averaged to evaluate the global strain, which included estimation of radial strain (L-RS) and longitudinal strain (L-LS) derived from the long-axis view, as well as radial strain (S-RS) and circumferential strain (S-CS) derived from the short-axis view. We also estimated global SR including radial strain rate (L-RSR) and longitudinal strain rate (L-LSR) from the long-axis view, as well as radial strain rate (S-RSR) and circumferential strain rate (S-CSR) from the short-axis view (Fig. 2). The reviewer (HK Yu) received specialized training evaluating the echocardiography data was blinded to the mice population.

### Estimation of intra‑observer and inter‑observer variability

Two observers independently analyzed ten randomly selected mice (5 mdx and 5 control mice) at various times and the intra- and inter-observer variability of the strain parameters were estimated. To determine intra-observer variability, the first observer analyzed the data twice with an interval of 2 weeks while being blinded to the results of the first set of measurements. The second observer was blinded to the results of the first observer and analyzed the data independently to determine the inter-observer variability. The intra- and inter-observer variability were expressed as intra-class correlation co-efficient (ICCs).

### Biochemical and histopathological examination

Peripheral blood samples were collected from all the mice in each group and the serum levels of liver function parameters (alanine transaminase (ALT), aspartate transaminase (AST), alkaline phosphatase (ALP)) and myocardial zymography indicators (lactate dehydrogenase (LDH), creatine kinase (CK)) were estimated.

Myocardial tissue samples were analyzed by immunofluorescence staining with the monoclonal anti-dystrophin antibody from Sanying Biotechnology Co., Ltd (Wuhan, China) and the content of positively-stained fibers were estimated for all the mice in the 3 groups. The integrated optical density (IOD) of five fields in each section and the corresponding five fields of tissue area were measured separately using Image-Pro Plus 6.0 software, then the mean optical density (MOD) was calculated by total IOD / total tissue area. MOD represented the dystrophin level. Isotype IgG was used as negative control in immunofluorescence staining.

### Statistical analysis

Statistical analysis was performed using the SPSS software version 23.0 (IBM Corp., Armonk, NY, USA) and the MedCalc software version 18.2.1 (MedCalc Software, Mariakerke, Belgium). The continuous variables were expressed as means ± standard deviation (SD). The group differences for the continuous variables were compared using one-way analysis of variance. Bonferroni adjustment method was used for pairwise comparison of the means when comparing the differences between multiple groups of data. Two-tailed P-values < 0.05 were considered statistically significant. The intra-observer and inter-observer variability was reported as the coefficient of variation, which were calculated by dividing the standard deviation by the mean and expressed as a percentage. The intra- and inter-observer variabilities were expressed as percentiles, which were calculated as follows: (absolute difference between the measurements/mean of the measurements) ×100%.

## Results

### Traditional echocardiography parameters are similar in all 3 groups of mice

All the mice underwent high-frequency echocardiography examinations as well as biochemical and immunofluorescence examinations. Traditional echocardiography parameters such as LVEDD, LV mass, LVEF, LVFS, and HR did not show any significant differences in the 3 groups of mice (Table 1).

**Table 1:** Traditional echocardiography parameters between BMSCs-transplanted mdx, untreated mdx and control mice

	BMSCs-transplanted mdx	Untreated mdx	Control mice	***F***	***P***
LV mass (mg)	115.2 ± 10.6	124.2 ± 22.7	108.1 ± 18.4	1.201	0.328
LVEDD (ml)	3.87 ± 0.13	4.06 ± 0.30	3.75 ± 0.30	2.241	0.141
LVEF (%)	73.84 ± 7.26	73.85 ± 3.72	76.06 ± 5.88	0.291	0.752
LVFS (%)	42.65 ± 6.91	42.48 ± 3.21	44.4 ± 5.45	0.231	0.796
HR (bpm)	355.5 ± 32.3	356.2 ± 13.4	375.7 ± 32.7	1.031	0.381

* F*: the statistical value of analysis of variance

### BMSCs-transplanted mdx show significant improvements in the two-dimensional STE parameters

**Fig. 3:**
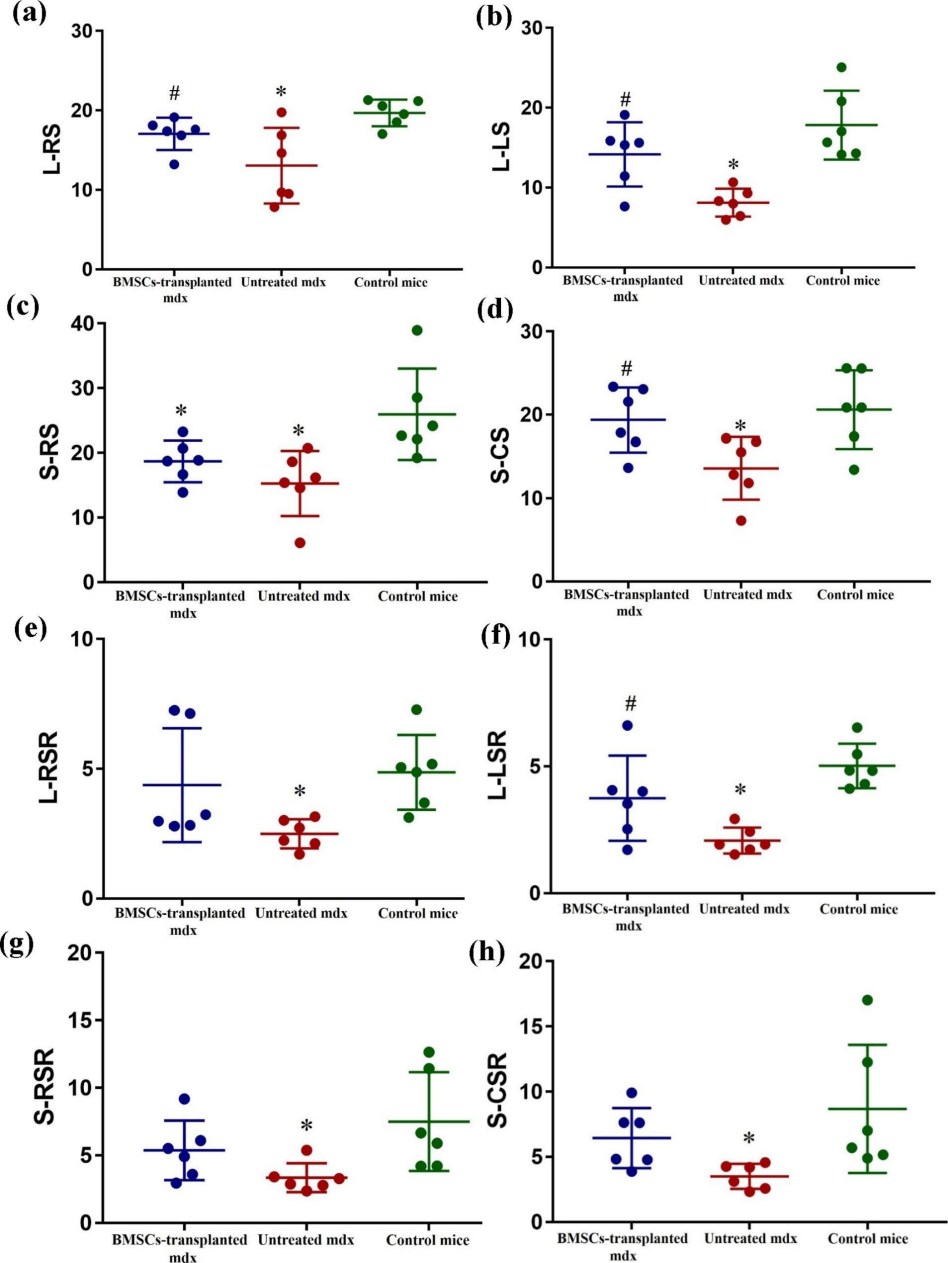
Comparation of 2D STE parameters including L-RS(a), L-LS(b), S-RS(c), S-CS(d), L-RSR(e), L-LSR(f), S-RSR(g), and S-CSR(h) between BMSCs-transplanted mdx, untreated mdx and control mice. *P < 0.05 vs. control, #*P* < 0.05 vs. untreated mdx

Table 2 shows the 2D STE parameters for the 3 groups of mice. Comparation of the global strain (L-RS, L-LS, S-RS, S-CS) and the global SR (L-RSR, L-LSR, S-RSR, S-CSR) for the 3 groups of mice are shown in Fig. 3.

**Table 2:** Comparison of 2D STE parameters between BMSCs-transplanted mdx, untreated mdx and control mice

	BMSCs-transplanted mdx	Untreated mdx	Control mice	***F***	***P***
L-RS (%)	17.05 ± 2.03^b^	12.48 ± 3.94^a^	19.69 ± 1.68	6.798	0.008
L-LS (%)	14.17 ± 4.02^b^	8.11 ± 1.76^a^	17.83 ± 4.30	11.479	0.001
S-RS (%)	18.67 ± 3.23^a^	15.26 ± 5.03^a^	25.94 ± 7.06	6.264	0.011
S-CS (%)	19.38 ± 3.90^b^	13.58 ± 3.75^a^	20.62 ± 4.72	4.939	0.022
L-RSR (%/s)	4.37 ± 2.19	2.49 ± 0.56^a^	4.87 ± 1.44	3.919	0.043
L-LSR (%/s)	3.74 ± 1.68^b^	2.08 ± 0.52^a^	5.02 ± 0.88	10.17	0.002
S-RSR (%/s)	5.37 ± 2.21	3.34 ± 1.06^a^	7.50 ± 3.66	4.009	0.040
S-CSR (%/s)	6.44 ± 2.31	3.50 ± 0.96^a^	8.67 ± 4.92	3.977	0.041

^a^ Statistically significant compared with control mice

^b^ Statistically significant compared with untreated mdx

* F*: the statistical value of analysis of variance

The mdx showed significantly lower LV global strain in both the long-axis and the short-axis LV images compared with the control mice (*P* < 0.05). Furthermore, mdx transplanted with BMSCs demonstrated significantly higher RS and LS in the long-axis LV images and CS in the short-axis images compared with the untreated mdx (*P* < 0.05). However, RS in the short-axis images did not show statistically significant differences between the untreated mdx and the BMSCs-transplanted mdx. Except RS in the short-axis in BMSCs transplanted mdx showed significant reduction than RS in control mice (*P* < 0.05), there were no significant difference in the other LV global strain values between the control mice and the BMSCs-transplanted mdx.

In the long axis and short axis LV images of all the LV segments, the LV SR was significantly reduced in the mdx compared with the control mice (*P* < 0.05). SR values were higher in all the LV segments of the BMSCs-transplanted mdx compared with the untreated mdx. However, only the longitudinal SR values in the long-axis images were significantly higher in the BMSCs-transplanted mdx compared with the untreated mdx (*P* < 0.05). There was no significant difference between the control mice and the BMSCs-transplanted mdx in all the LV global SR values.

### STE parameters showed significantly high intra- and inter-observer reproducibility

The intraclass correlation coefficients (ICCs) for the intra- and inter-observer variability in the 2D strain parameters including L-RS, L-LS, S-RS, S-CS, L-RSR, L-LSR, S-RSR, and S-CSR were in the range of 0.868–0.916 (all *P* values < 0.001).

### Biochemical findings

The mdx showed significantly higher ALT, AST, CK, and LDH compared with the control mice (*P* < 0.05). There was no significant difference in ALP between the mdx and the control mice. The mdx transplanted with BMSCs showed significantly lower AST compared with the untreated mdx (*P* < 0.05), and there was no significant difference between the two groups in the other biochemical parameters. Compared with the control mice, the mdx transplanted with BMSCs showed significantly higher ALP, CK, and LDH (*P* < 0.05), and there was no significant difference between the two groups in ALT and AST. (See Table 3).

**Table 3:** Comparison of hepatic enzymes and cytokines levels between BMSCs-transplanted mdx, untreated mdx and control mice

	BMSCs-transplanted mdx	Untreated mdx	Control mice	***F***	***P***
ALT (U/L)	263.0 ± 195.1	463.5 ± 277.9^a^	55.33 ± 20.3	6.480	0.009
AST (U/L)	580.7 ± 297.6 ^b^	2676.0 ± 2096.3^a^	132.0 ± 113.4	7.383	0.006
ALP (U/L)	98.17 ± 19.29 ^a^	86.83 ± 15.01	66.83 ± 16.35	5.238	0.019
CK (U/L)	2294.0 ± 1721.3^a^	3129.0 ± 909.6^a^	403.5 ± 313.2	9.027	0.003
LDH (U/L)	4351.0 ± 2397.8^a^	4827.7 ± 1541.9^a^	1326.0 ± 387.2	7.843	0.005

^a^ Statistically significant compared with control mice

^b^ Statistically significant compared with untreated mdx

* F*: the statistical value of analysis of variance

### BMSCs-transplanted mdx show significantly improved myocardial dystrophin expression

**Fig. 4:**
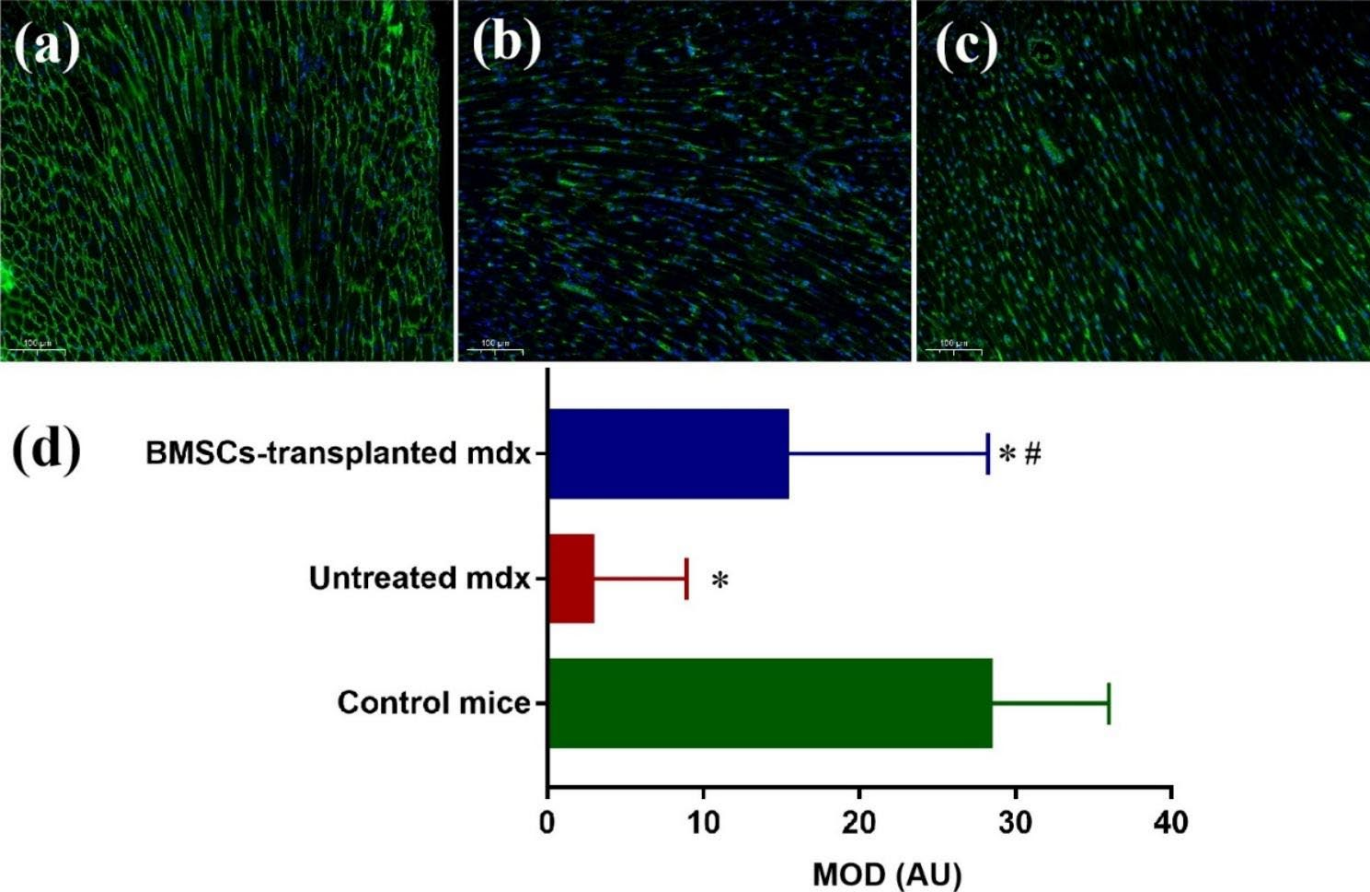
Representative photomicrographs of dystrophin immunofluorescence staining (magnification ×200; scale bar 100 μm) of myocardial fiber. (a) control mice; (b) untreated mdx; (c) BMSCs-transplanted mdx; (d) comparation of MOD among three groups. *P < 0.05 vs. control, #*P* < 0.05 vs. untreated mdx. AU: Arbitrary Units

The immunofluorescence staining results showed that the levels of dystrophin were significantly reduced in the untreated mdx compared to the control group, but increased significantly in the BMSCs-transplanted mdx. The untreated mdx showed significantly lower MOD compared with the control mice (*P* < 0.05). The mdx transplanted with BMSCs demonstrated significantly higher MOD compared with the untreated mdx (*P* < 0.05) (Table 4; Fig. 4).

**Table 4:** Difference of myocardial dystrophin expression between BMSCs-transplanted mdx, untreated mdx and control mice

	BMSCs-transplanted mdx	Untreated mdx	Control mice	***F***	***P***
MOD (AU)	15.28 ± 12.97^ab^	2.84 ± 6.05^a^	28.32 ± 7.68	11.080	0.001

^a^ Statistically significant compared with control mice

^b^ Statistically significant compared with untreated mdx

AU: Arbitrary Units

* F*: the statistical value of analysis of variance

## Discussion

This is the first study to investigate changes in the cardiac function of mdx after transplantation of BMSCs using high-frequency ultrasound-based two-dimensional STE. The analysis of STE parameters showed myocardial deformation in the mdx. However, conventional M-mode echocardiography did not detect any early LV contractile dysfunction in the mdx. The histopathology and STE parameters showed significant improvements in the myocardial structural parameters of the BMSCs-transplanted mdx compared to the untreated mdx. To our knowledge, this is the first study that used 2D strain and strain rate (SR) values derived from STE to evaluate the impairment of systolic and diastolic function in the hearts of the mdx, and detect changes in myocardial function before and after transplantation of the BMSCs. Our study demonstrated that the BMSCs transplantation therapy improved cardiac function in the mdx, and changes in the cardiac function of mdx after BMSCs transplantation can be detected early by estimating 2D strain and SR using high-frequency ultrasound-based two-dimensional STE. The present study suggested that BMSCs transplantation therapy may be promising for patients with DMD.

Our results showed that the myocardial strain and SR derived from STE were significantly reduced in the mdx compared with the control mice. However, the cardiac contractile function parameters such as LVEF and LVFS, which were measured by conventional M-mode echocardiography, did not show any significant differences between the two groups. Therefore, our study confirmed that strain and SR, which reflect myocardial exercise capacity, can be used to detect changes in the myocardial function under pathological conditions. Although M-mode echocardiography-derived parameters such as LVEF, LVFS, LV mass and chamber diameter can be used to evaluate cardiac function and morphology, many previous studies have confirmed that 2D strain is a more sensitive measure of cardiac dysfunction than the conventional M-mode echocardiography parameters in patients with DMD [21, 22] and the mdx [18, 23]. We previously demonstrated that 3D strain can be used to detect cardiac dysfunction in children with DMD [16]. The present study confirmed the sensitivity of strain in detecting cardiac in the mdx and the results were consistent with clinical studies regarding myocardial strain changes in the young DMD patients [24]. Therefore, our results further support the use of strain parameters to detect early LV dysfunction in DMD patients. Previous studies in rats have shown that abnormal radial strain is associated with histological changes in the myocardium [25]. Li et al. [18] also used strain in combination with stress ultrasound to evaluate cardiac function in the mdx and control mice, but only examined strain and did not evaluate SR. The results of this study showed significantly reduced SR in the mdx compared to the control mice. This demonstrated that in addition to reduced systolic function, diastolic function was also altered during myocardial injury in the mdx. This finding suggests that the therapeutic interventions to improve cardiac function in children with DMD must focus on restoring both systolic and diastolic functions.

STE is a useful technique to detect myocardial deformation by estimating epicardial strain, endocardial strain and other myocardial parameters [23]. In the present study, we traced the entire LV wall from the epicardial to the endocardial border because cardiac muscle degeneration is widely involved in the pathology of heart dysfunction in the mdx [26, 27]. However, previous studies demonstrated structural and functional differences between endocardium and epicardium; the contribution of endocardium towards cardiac contractile motion was significantly higher than the epicardium [28, 29]. Endocardial strain is more sensitive than the epicardial strain in distinguishing the mdx from the control mice [18].

Several studies have shown that MSCs can be differentiated into cardiomyocyte-like cells [30]. Furthermore, MSCs can evade immune detection, secrete a range of anti-inflammatory and anti-fibrotic mediators, and activate heart-resident cardiomyocyte precursors [31]. These features form the basis for the clinical application of MSCs by transplantation in patients with ischemic and non-ischemic cardiomyopathies and have shown promising results [31]. Many preclinical studies have demonstrated that the transplantation of MSCs reduces fibrosis, stimulates angiogenesis, and improves remodeling of the ventricular structure and function, which is evident in the ischemic and non-ischemic cardiomyopathies [32, 33]. Klimczak et al. [34] reported that the transplantation of BMSCs and stem/progenitor cells (SM-SPCs) derived from the skeletal muscle into DMD patients improved their muscle function and dystrophin expression without adverse effects. BMSCs ameliorate remodeling of ventricular structure and function resulting from ischemic and non-ischemic cardiomyopathies by reducing myocardial fibrosis and stimulating angiogenesis [35]. The results of our study were consistent with previous results. Our study showed alleviation of myocardial deformation in the BMSCs-transplanted mdx. Although some parameters did not show statistically significant differences between the untreated and BMSCs-transplanted mdx, all the strain and SR measurements showed distinct improvements in the BMSCs-transplanted mdx. The small sample size might be one reason for some of the parameters not showing statistically significant differences. Furthermore, SR may be susceptible to changes in factors such as heart rate.

In the present study, we also confirmed the amelioration due to BMSC transplantation in the cardiac function and structure of the mdx by biochemical and histopathological examinations. The levels of dystrophin were significantly reduced in the untreated mdx compared to the control group, but increased significantly in the BMSCs-transplanted mdx. Alterations of hepatic enzymes and myocardial zymography indicators in DMD are a response that results from long-term damage to muscle. Although damage of muscle tissue was ameliorated after transplantation of BMSCs and enzyme levels decreased substantially, we considered that there were no statistical differences because of the small sample size and limited follow-up time. This reflected to a certain extent that biochemical indicators were not sensitive in evaluating the efficacy of BMSCs transplantation.

The coupling of muscle progenitor cell proliferation with myogenic differentiation is necessary to improve myocardial function by transplanting BMSCs. Previous reports have shown the potential of BMSCs to fuse and form myotubes, and the ability of BMSCs to promote tissue regeneration [34, 36]. Furthermore, BMSCs alter the immune regulatory microenvironment by secreting immunosuppressive cytokines [34]. Muscle degeneration in DMD is accompanied by chronic inflammation due to overexpression of pro-inflammatory cytokines [37, 38]. Therefore, inflammatory response is a major source of excessive cytokines that promote cytolytic and cytotoxic effects on the myofibers [39, 40]. BMSCs promote modification of the inflammatory M1 type macrophages with pro-inflammatory, anti-angiogenic, and tissue growth inhibitor effects into the M2 phenotype with anti-inflammatory, pro-remodeling, and tissue healing effects. which are necessary for muscle regeneration [41]. Modulation of inflammation is beneficial for DMD, and BMSCs are considered as anti-inflammatory agents for the treatment of DMD [42].

## Limitations of this study

This study has a few limitations. Firstly, echocardiography was performed under anesthesia, which can affect myocardial contractility, ejection fraction and strain/SR values. Secondly, the treatment course of BMSCs transplantation in this study was short. In the future, long-term efficacy of the BMSCs needs to be investigated by increasing the number of BMSCs transplantations and prolonging the time course. Thirdly, the sample size was small in the present study. Therefore, in the future studies, a large sample size is required to confirm our results. Finally, this study was performed in an animal model of DMD (mdx), which does not entirely represent the same disease process as the human DMD patients. Therefore, our study needs to be validated in the DMD patients in future studies.

## Conclusion

This study demonstrated that the 2D STE technique detected early reduction in the LV systolic and diastolic functions of the mdx. Furthermore, this study confirmed that transplantation of BMSCs significantly improved myocardial function in the DMD model mdx. Future experiments in animal models and human trials in DMD patients are necessary to confirm that the transplantation of BMSCs is a safe and effective clinical treatment for DMD.

## Data Availability

All data supporting the findings of this study are available within the published article.

## Abbreviations

BMSCs: Bone marrow mesenchymal stem cells
STE: speckle tracking echocardiography
LV: left ventricular
DMD: Duchenne muscular dystrophy
MSCs: Mesenchymal stem cells
EF: ejection fraction
FS: fractional shortening
LVEDD: LV end-diastolic dimension
HR: heart rate
ROIs: regions of interest
SR: strain rate
RS: radial strain
LS: longitudinal strain
CS: circumferential strain
RSR: radial strain rate
LSR: longitudinal strain rate
CSR: circumferential strain rate
ICCs: intra-class correlation co-efficient
ALT: alanine transaminase
AST: aspartate transaminase
ALP: alkaline phosphatase
LDH: lactate dehydrogenase
CK: creatine kinase
IOD: Integrated optical density
MOD: Mean optical density
SD: standard deviation
