# Correction: Two-dimensional speckle tracking echocardiography demonstrates improved myocardial function after intravenous infusion of bone marrow mesenchymal stem in the X-Linked muscular dystrophy mice

**DOI:** 10.1186/s12872-022-02949-3

**Published:** 2022-11-29

**Authors:** Xiao Liu, Shixiang Yao, Min Pan, Yingying Cai, Weihui Shentu, Wenqian Cai, Hongkui Yu

**Affiliations:** 1 Department of Ultrasonography, Shenzhen Hospital of Guangzhou University of Chinese Medicine (Fu-tian), Shenzhen, Guangdong China; 2 Department of Ultrasonography, Guangzhou Women and Children’s Medical Center, Guangzhou Medical University, Guangzhou, Guangdong China; 3 Heart Center, Institute of Pediatrics, Guangzhou Women and Children’s Medical Center, Guangzhou Medical University, Guangzhou, Guangdong China; 4 Department of Ultrasonography, Shenzhen Children’s Hospital, Shenzhen, Guangdong China

**Correction: BMC Cardiovasc Disord 22, 461 (2022)**

**https://doi.org/10.1186/s12872-022-02886-1**

Following the publication of the original article [1], the affiliation of the corresponding author has been swapped and it should read as follows:

Xiao Liu^1†^, Shixiang Yao^2†^, Min Pan^1^, Yingying Cai^2^, Weihui Shentu^2^, Wenqian Cai^3^ and Hongkui Yu^4,2*^

Author Xiao Liu also equally contributed to this article and it should read as follows:

^†^Shixiang Yao and Xiao Liu has equally contributed.

The original article has been corrected.
